# Denaturing Gradient Gel Electrophoresis Approach for Microbial Shift Analysis in Thermophilic and Mesophilic Anaerobic Digestions

**DOI:** 10.3390/gels10050339

**Published:** 2024-05-16

**Authors:** Pramod Pandey, Dhrubajyoti Chowdhury, Yi Wang

**Affiliations:** 1 Department of Population Health and Reproduction, University of California-Davis, Davis, CA 95616, USA; dchowdhu@gitam.edu (D.C.); yyiwang@ucdavis.edu (Y.W.); 2 Department of Life Sciences, School of Science, Gandhi Institute of Technology and Management, Rushikonda, Visakhapatnam 530045, Andhra Pradesh, India

**Keywords:** gel electrophoresis, denaturing, sequencing, PCR, anaerobic digestion

## Abstract

To determine the evolution of microbial community and microbial shift under anaerobic processes, this study investigates the use of denaturing gradient gel electrophoresis (DGGE). In the DGGE, short- and medium-sized DNA fragments are separated based on their melting characteristics, and this technique is used in this study to understand the dominant bacterial community in mesophilic and thermophilic anaerobic digestion processes. Dairy manure is known for emitting greenhouse gases (GHGs) such as methane, and GHG emissions from manure is a biological process that is largely dependent on the manure conditions, microbial community presence in manure, and their functions. Additional efforts are needed to understand the GHG emissions from manure and develop control strategies to minimize the biological GHG emissions from manure. To study the microbial shift during anaerobic processes responsible for GHG emission, we conducted a series of manure anaerobic digestion experiments, and these experiments were conducted in lab-scale reactors operated under various temperature conditions (28 °C, 36 °C, 44 °C, and 52 °C). We examined the third variable region (V3) of the 16S rRNA gene fingerprints of bacterial presence in anaerobic environment by PCR amplification and DGGE separation. Results showed that bacterial community was affected by the temperature conditions and anaerobic incubation time of manure. The microbial community structure of the original manure changed over time during anaerobic processes, and the community composition changed substantially with the temperature of the anaerobic process. At Day 0, the sequence similarity confirmed that most of the bacteria were similar (>95%) to *Acinetobacter* sp. (strain: ATCC 31012), a Gram-negative bacteria, regardless of temperature conditions. At day 7, the sequence similarity of DNA fragments of reactors (28 °C) was similar to *Acinetobacter* sp.; however, the DNA fragments of effluent of reactors at 44 °C and 52 °C were similar to *Coprothermobacter proteolyticus* (strain: DSM 5265) (similarity: 97%) and *Tepidimicrobium ferriphilum* (strain: DSM 16624) (similarity: 100%), respectively. At day 60, the analysis showed that DNA fragments of effluent of 28 °C reactor were similar to *Galbibacter mesophilus* (strain: NBRC 10162) (similarity: 87%), and DNA fragments of effluent of 36 °C reactors were similar to *Syntrophomonas curvata* (strain: GB8-1) (similarity: 91%). In reactors with a relatively higher temperature, the DNA fragments of effluent of 44 °C reactor were similar to *Dielma fastidiosa* (strain: JC13) (similarity: 86%), and the DNA fragments of effluent of 52 °C reactor were similar to *Coprothermobacter proteolyticus* (strain: DSM 5265) (similarity: 99%). To authors’ knowledge, this is one of the few studies where DGGE-based approach is utilized to study and compare microbial shifts under mesophilic and thermophilic anaerobic digestions of manure simultaneously. While there were challenges in identifying the bands during gradient gel electrophoresis, the joint use of DGGE and sequencing tool can be potentially useful for illustrating and comparing the change in microbial community structure under complex anaerobic processes and functionality of microbes for understanding the consequential GHG emissions from manure.

## 1. Introduction

In anaerobic environment, microbial community structure plays an essential role in controlling the organic carbon degradation, during which larger molecules are broken down into smaller molecules, and intermediate products such as hydrogen and volatile fatty acids and final products such as methane are produced. In order to know these anaerobic degradation processes, understanding the microbial community structure during the anaerobic process and consequential product formation under different temperature conditions is critically important, and it is yet to be fully understood. In the context of global warming and climate change, a substantial emphasis is given to problems of greenhouse gas (GHG) emissions from animal waste, and the demand for technology capable of controlling GHG emissions from manure has increased substantially. In this study, we evaluated DNA fragments of effluents of anaerobic digesters by the denaturing gradient gel electrophoresis (DGGE) technique for determining the microbial community structures under different environmental conditions. 

**Figure 1:**
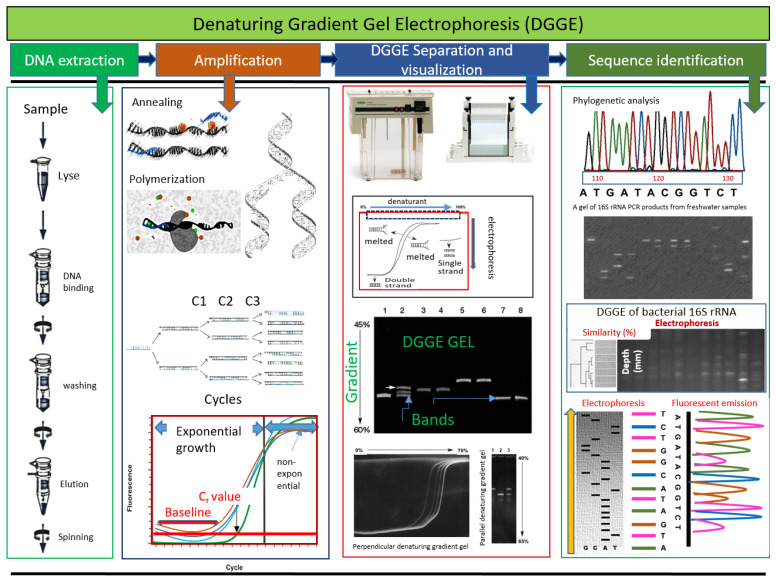
Conceptual workflow diagram showing different steps involved in the PCR-DGGE-Sequencing approach to study the structure of microbial community and the format and visualization of results. In the DGGE approach, genetic fingerprinting by DGGE is crucial to investigate the bacterial communities in complex biomaterials such as dairy manure. This conceptual workflow resulted from reviewing and redrawing of the previous work [14,15,16,17,18,19,20].

The DGGE technique allows for a rapid analysis of multiple samples and provides temporal and spatial dynamics of microbial communities based on experiment and treatment designs [1,2]. While originally the DGGE gel approach was formulated to understand the single-nucleotide polymorphisms in genes [2], currently this technique is used in various fields including environmental microbiology and microbial ecology [3,4,5]. In general, the working principle of DGGE is based on the partial DNA strand separation at a particular position in a gradient of chemical denaturant [2]. DGGE uses polyacrylamide gels with an increasing gradient of chemical denaturants (such as urea and formamide), and DNA molecules are passed through the gel by electrophoresis. In case, when a double-stranded DNA passes through these chemical gradients, each molecule starts to denature at a unique concentration of denaturant. The gradients of denaturant are run parallel to the direction of electrophoresis, and bands are observed at locations where individual molecules partially denature (Figure 1) due to the gradients of denaturant [2,4]. In essence, the DGGE gel-based approach is a nucleic acid separation technique that allows biodiversity evaluation. One of the main advantages of the DGGE-based approach is the ability to evaluate various DNA fragments in a single gel using denaturing gradient, and while the application of this technique is relatively complex, it is very useful in various settings. The DGGE gel technique can separate medium-to-short lengths of DNA fragments based on their melting characteristics, and it can even be used in identifying single-nucleotide polymorphisms without the need for DNA sequencing [2,4]. 

In understanding the role of microbiology during organic matter degradation, the development and application of gel electrophoresis as a method for separation and analysis of DNA fragments have been one of the driving forces. The emission of GHGs such as methane during anaerobic microbial degradation is a complex process, and it is poorly understood in terms of determining the GHG emissions from livestock waste. To understand the role of microbes in GHG emissions, the separation, identification, and analysis of DNA fragments are crucial, and there are multiple recent advances to improve the existing understanding of the structure and function of microbes and biological methane emissions [6,7,8]. Considering livestock waste is a significant source of GHG emission, additional research is needed to develop pragmatic approaches for on-farm manure management. Currently, an enormous amount of dairy manure is produced in dairy industry, and subsequently, this manure is processed through anaerobic and composting processes to convert manure into soil amendments [9,10,11]. While ruminal microbiota present in dairy manure supports useful anaerobic biodegradation processes, during anaerobic digestion, many additional bacterial communities including methanogenic community produce GHGs such as methane [9,12]. 

In order to minimize GHG emissions from livestock manure, development and application of improved on-farm manure management techniques are needed. There are many manure management practices (i.e., composing, drying, anaerobic digestion), and to enhance the impacts of these practices on controlling methane emissions, improved understanding of bacterial communities responsible for these emissions during on-farm manure management are crucial. When it comes to knowing the bacteria in livestock manure, there are multiple methods such as most probable number (MPN), qPCR, clone library, fluorescence in situ hybridization (FISH), pyrosequencing, DNA microarray, stable isotope probing, and RT-qPCR, which are used. These methods allow for understanding microbial community structures and functions [13]. Often, the small subunit ribosomal RNA sequences and the potential of phylogenetic analysis are utilized to determine the microbial diversity [1]. However, the implementation of these microbial testing methods to determine the microbial community structure in complex organic matter such as livestock manure is often challenging. 

Combining the strength of DGGE with a sequencing approach could reveal additional insights to improve understanding of microbial community and consequential GHG emissions. A general workflow diagram is illustrated in Figure 1, indicating various steps involved in PCR-DGGE-Sequencing. 

In the DGGE technique, PCR primers with GC clamps (35–40 nt GC-rich tails) are used, which allows the partial melting of structure where GC clamp remains double stranded [2]. DGGE is particularly useful for molecular fingerprinting to assess microbial diversity in complex samples with a mixed microbial community [2,4]. DGGE allows the separation of the DNA fragments of the same size and different sequence, which assists in understanding bacterial diversity in environmental samples such as soil [4]. The use of the DGGE gel approach is explored previously on processed waste to target typical bacteria of stabilized organic waste, and the approach was able to provide distinctive microbial fingerprints of different waste material, and the combination of DGGE with COMPOCHIP (a microarray) showed the presence of *Sphingobacterium*, *Streptomyces*, Alpha-Proteobacteria, Delta-Proteobacteria, and Firmicutes in vermicomposting [3]. The application of DGGE genetic fingerprinting in the marine microbial ecology was found to be useful for monitoring variability in microbial genetic diversity, and the DGGE technique facilitated the identification of individual populations [21]. 

In order to understand the dynamics of microbial community in swine manure during long-term storage, a previous study used the PCR-DGGE of 16S rDNA approach, and various phylotypes were identified by excising and cloning DGGE bands and comparing the 16S rDNA sequence with the sequence available in GenBank [22]. The DGGE gel technique used in this study illustrated the presence of *Clostridium butyricum*, *Clostridium disporicum*, *Pedobacter* sp., and *Rhodanobacter* sp. in swine manure. Further, the PCR-DGGE method was useful in assessing the bacterial diversity of treated and untreated milk during cold storage, and the PCR-DGGE profile showed the presence of various dominant bacteria including *Streptococcus*, *Staphylococcus*, *Pseudomonas*, *Aerococcus*, *Trichococcus floculiformis,* and *Prevotella* spp. [23]. Previous research showed that fingerprinting techniques such as DGGE provide the best compromise when it comes to the number of samples processed and the amount of information generated [1]. Considering our complex experiment and the number of temperature conditions and incubation periods used in this study, we utilized the potential of DGGE to understand the microbial dynamics in anaerobic digestion processes. 

In this study, we used the PCR-DGGE-Sequencing method to understand microbial community evolution based on DNA fragments under anaerobic processes. The main objectives of this study were to determine the similarity and type of microbial communities present in mesophilic and thermophilic anaerobic processes. In real-world anaerobic digestion system treating waste, there are about four phases: (1) startup phase (which varies from 5 to 10 days); (2) intermediate phase (10–15 days of retention time); (3) commonly used retention time in a full-scale system (30–40 days); and (4) late digestion phase (50–60 days). In this study, we extracted the samples from these stages to understand the microbial shift. In addition, both mesophilic and thermophilic digestions are used for degrading biomass anaerobically. Therefore, this study involves investigating both thermophilic and mesophilic anaerobic digestion processes. To examine the impacts of temperature on microbial communities, a series of anaerobic experiments were conducted at various temperature levels using dairy manure as feedstock. Further, to test the impacts of incubation time on the evolution of microbial community, effluent samples were collected at various retention times from anaerobic reactors. Overall, the goal of this study was to enhance our understanding of microbial community changes in anaerobic processes by using the PCR-DGGE-Sequence-based approach. This approach was tested in effluents obtained from lab-scale anaerobic reactors operating under mesophilic and thermophilic conditions (28–52 °C). During experiments, samples were collected from reactors at various incubation times, and subsequently, genomic DNA was extracted from the effluent. This genomic DNA was amplified using PCR, and DGGE gel-based separation and visualization were performed. Subsequently, the characteristics of bands obtained under various temperature and incubation conditions were further analyzed through the sequencing process. 

## 2. Results and Discussion

### 2.1. PCR-DGGE Analysis of Initial Samples

**Figure 2:**
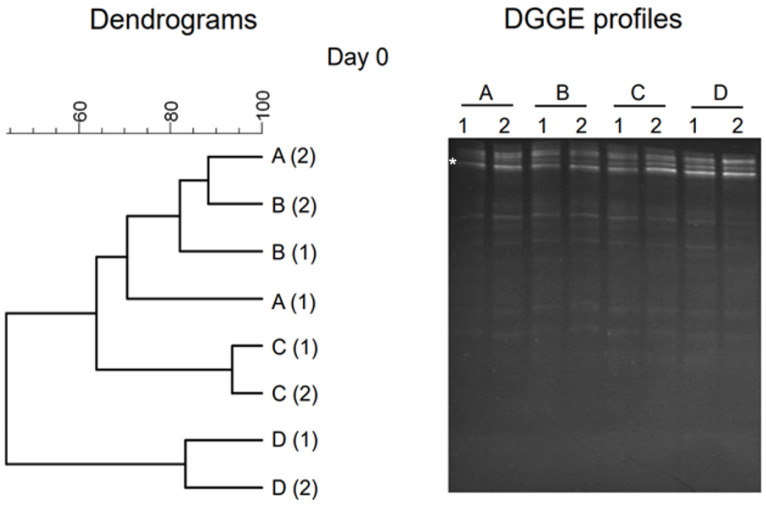
Results of PCR-DGGE analysis in initial sample (Day 0). Dendrograms are shown on the left, and the DGGE gel profile is shown on the right. Numbers (1, 2) represent samples, and letters (A, B, C, D) represent temperature conditions (28 °C, 36 °C, 44 °C, and 52 °C). Bands excised for sequencing are marked (*), and sequencing results are shown in Table 1.

The bacterial community of the initial feedstock used in anaerobic process was evaluated by analyzing the initial samples, and the DGGE profile and dendrograms shown in Figure 2 indicate the locations of bands and similarity. The cluster analysis for bacteria shows the similarity of initial samples among all reactors at Day 0. DGGE profiles show that a majority of the bands of Day 0 samples were located at the top of the gel, and the similarity in reactors within treatment was relatively higher. The results of cluster analysis of the DNA-based DGGE gel revealed that each reactor had a slightly similar bacterial community in the initial stage of experiments because of the start-up stage. In the DGGE profile, a smaller cluster indicates the smaller community shifts during cluster analysis [24]. DGGE patterns showed a relatively low coverage (i.e., distribution of bands), which can be due to the initial stage of samples. DGGE profiles and dendrograms showed that bands of samples A (28 °C) and B (36 °C) had a relatively higher level of similarity than that of samples C (44 °C) and D (52 °C). Similarly, bands of samples C and D were more similar than bands of samples A and B. 

### 2.2. Microbial Dynamics in Intermediate Stages of Anaerobic Processes

**Figure 3:**
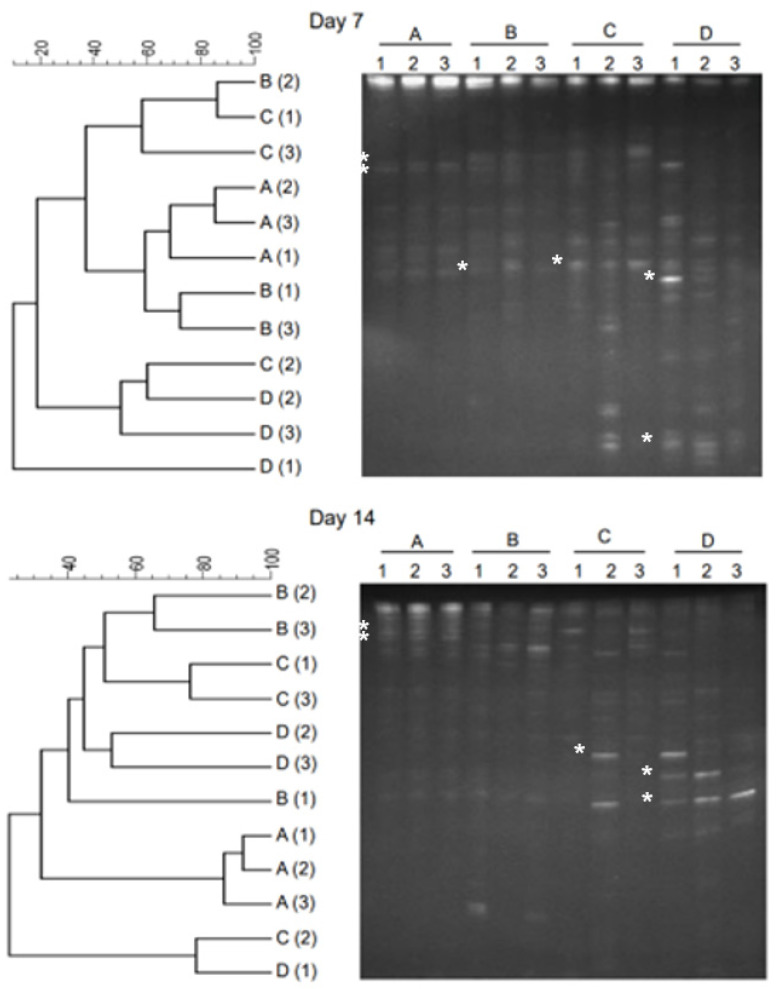
The results of PCR-DGGE analysis of anaerobic effluent during the startup phase (Day 7) and intermediate phase (Day 14). Dendrograms are shown on the left, and the DGGE gel profile is shown on the right. Numbers (1, 2) represent samples, and letters (A, B, C, D) represent temperature conditions (28 °C, 36 °C, 44 °C, and 52 °C). Bands excised for sequencing are marked (*), and sequencing results are shown in Table 1.

Figure 3 showed the DGGE profile and dendrogram clustering of samples collected on Day 7 and Day 10. Compared to the initial stage of samples (Day 0), the samples collected at Day 7 and Day 14 showed a higher level of coverage. The bands were more dispersed within the lane (Figure 3). DGGE patterns showed that this dispersion in bands was higher in the thermophilic temperature (C and D) range than in the mesophilic temperature range (A and B). The highest coverage was obtained in samples C and D, obtained from reactors operated under thermophilic temperatures. The dendrogram clustering shows (Figure 3) that samples of reactor operated under mesophilic temperatures (28 °C and 36 °C) were relatively similar (higher level of similarity). However, reactors with thermophilic temperatures (44 °C and 52 °C) showed less similarity to the samples from lower temperatures. Reactors operated within the same temperature conditions showed higher level of similarity. While comparing Day 7 and Day 0 samples, the similarity varied between 19% and 22% at 28 °C. However, there was no similarity at 36 °C, 44 °C, and 52 °C. Comparison between Day 14 and Day 7 samples showed that, at 28 °C, the similarity varied between 29 and 43%. At 36 °C, the similarity varied between 0 and 15%. In the thermophilic temperature range, the similarity was substantially less. For example, at 44 °C, the similarity varied between 0 and 16%, and at 52 °C, the similarity varied between 0 and 9%. 

### 2.3. Microbial Dynamics in Late Stages of Anaerobic Processes

**Figure 4:**
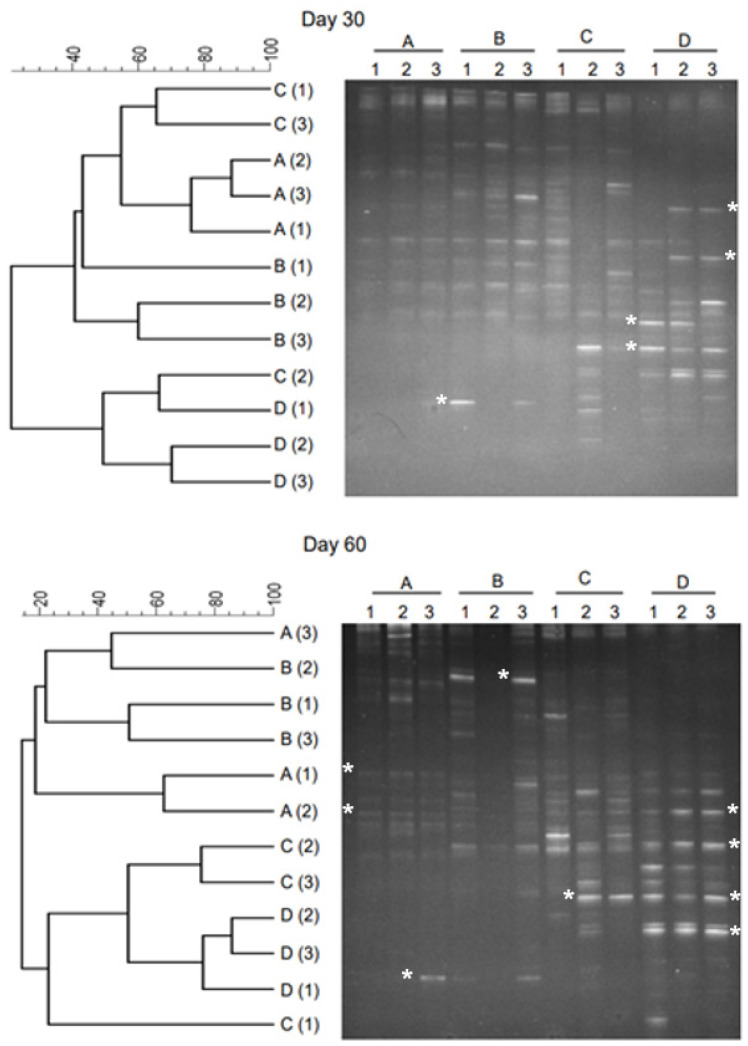
PCR-DGGE analysis results of the post-anaerobic digestate. Effluent samples at Day 30 and Day 60 are considered as digestate of anaerobic digester. In general, anaerobic digester retention time is 30–40 days. Dendrograms are shown on the left, and the DGGE gel profile is shown on the right. Numbers (1, 2) represent samples, and letters (A, B, C, D) represent temperature conditions (28 °C, 36 °C, 44 °C, and 52 °C). Bands excised for sequencing are marked (*), and sequencing results are shown in Table 1.

Microbial dynamics in the late stage of process is shown in Figure 4. The DGGE profile and dendrograms of samples collected on Day 30 and Day 60 are shown in the figure. Compared to the initial stage of samples (Day 0) and intermediate stages (Day 7 and Day 14), the samples collected at Day 30 and Day 60 showed substantially larger number of bands in the DGGE gel. The coverage of bands in each lane of a sample was much greater than the samples collected at initial and intermediate stages of the anaerobic reactions. 

The bands were substantially more dispersed and brighter within each lane (Figure 4). Similar to the samples from intermediate stages, the DGGE patterns of late stages showed that the dispersion was higher at thermophilic temperatures (A and B) than that at mesophilic temperatures (C and D). The highest coverage was obtained in samples C and D. The dendrogram clustering shows (Figure 4) that samples of reactor operated under mesophilic temperatures (28 °C and 36 °C) were relatively similar. The replicate reactors operated under the same temperature conditions showed higher level of similarity. While comparing Day 30 and Day 14 samples, the similarity varied between 24% and 39% at 28 °C. This similarity varied between 0% and 22% at 36 °C. In the thermophilic temperature range, the similarity varied between 0 and 32% for reactors at 44 °C, and 0 and 19% for reactors at 52 °C.

### 2.4. DGGE Profiles of Microbial Community Dynamics over Various Incubation Periods

**Figure 5:**
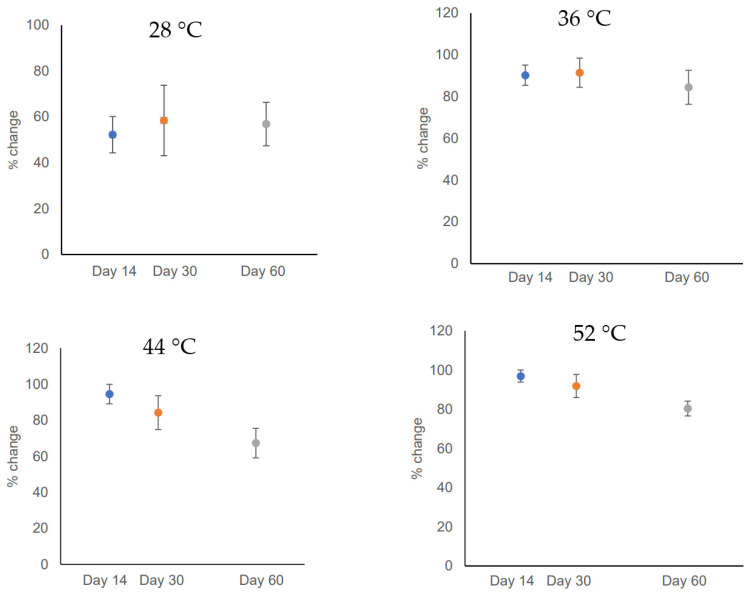
Microbial community dynamics based on DGGE profiles. The change in microbial community under mesophilic temperatures (28 °C and 36 °C) and thermophilic temperatures (44 °C and 52 °C) is shown.

Further analyses of DGGE patterns were conducted to estimate the dynamics of microbial communities, which are shown in Figure 5. This analysis shows the number of species that on average are of significant dominance over the various intervals of anaerobic processes. The rate of change parameter (i.e., % change) (Figure 5) was estimated based on moving window analysis, which provides unambiguous numerical measures to make comparison between results of DGGE profiles [25]. The rate of change parameter averages the degree of change between DGGE profiles of various incubation periods of the same treatment (i.e., same type of community) over the anaerobic process. As an example, the rate of change (%) shown for 28 °C compares the degree of change in reactors incubated at 28 °C for 60 days. The figure shows the rate of changes for Day 14, Day 30, and Day 60. This percentage change is estimated using the DGGE gel processing software, which allows to calculate similarities for the densiometric curves of the DGGE patterns based on Pearson product-moment correlation coefficients (% change = 100 − % similarity). These % change values are used to perform the moving window analysis by plotting the values between samples of various incubation periods (*x*-axis) and % change (*y*-axis). The higher changes between the DGGE profiles of two incubation points results in higher corresponding moving window curve data points and higher % change values [25]. The DGGE-based analysis provides the linkages between functional performance and the activity of specific microbial ecology. These techniques have been used previously to create DGGE profiles and estimate the corresponding rate of change in various treatment processes such as wastewater treatment reactors to understand the dynamics of ammonia-oxidizing bacteria [24,25]. 

Figure 5 shows that % change at a low temperature (28 °C) varied between 52% and 58%. When temperature of reactor was increased to 36 °C, the % change varied between 84% and 91%. Both 28 °C and 36 °C are considered to be in the mesophilic temperature range. In the thermophilic temperature range (44 °C and 52 °C), the % change values increased. For example, at 44 °C, the % change value varied between 67% and 94%. At 52 °C, the % change values varied between 80% and 96%. Results showed that, at a higher temperature (52 °C), the shifts in microbial community between Day 14 and Day 60 was the highest, followed by 44 °C, 36 °C, and 28 °C. In addition, analysis showed that the microbial community linearly declined in the thermophilic temperature range by the end of the 60-day incubation period, while in the mesophilic range, the direction of shifts in microbial communities was not clear as shifts in the thermophilic range. Based on DGGE profiles, reactors with thermophilic temperature yielded a different pattern than that of reactors with mesophilic temperatures. The trend was slightly similar at 28 °C and 36 °C; however, the % change reached 91% at 36 °C compared to 58% at 28 °C. This indicates the substantial shifts in microbial activity at 36 °C compared to those at a low temperature (28 °C). However, at both of these temperatures, the % change peaked in Day 30 samples. In contrast, the % change in thermophilic temperature peaked on Day 14, which indicates that the microbial activity in thermophilic conditions is accelerated, and it can reach a peak much earlier than that at a mesophilic temperature. These findings suggest that microbes can degrade the substrate at a much faster rate in the thermophilic temperature range because of increased microbial activity, and the required retention time of anaerobic digester can be reduced substantially (almost by 50%). The clustering and moving window analysis based on the DGGE gel profile were proven to be valuable to monitor microbial community shifts in the wastewater treatment process [24]. Previous studies showed that the % change in community is linked with the performance of treatment processes. As an example, the functionality of a microbial community in the nitrification process and the reactor performance were changed during wastewater treatment, when the moving window analysis of the DGGE profile showed fluctuations and instability [24]. 

### 2.5. Comparison of Bands, Sequence Sizes, Closest Relatives, and Alignment Similarities

A comparative analysis of extracted bands from different reactors, sequence sizes, accession numbers, and closest relatives are shown in Table 1. On Day 0 (initial samples), the DGGE profile showed a dominant band, and subsequently, this band was used for a downstream sequencing process. The numbers of bands extracted at each temperature over the incubation time are shown in Table 1. The sequence size varied between 169 and 195 bp over the incubation time. In the initial stage of experiment (Day 0), the sequence size was 195 bp. At Day 60, there were nine dominant bands, while in the start-up phase (Day 7), six bands were dominant. The number of extractable bands in the intermediate phases on Day 14 and Day 30 were five. During each incubation period, the dominant bands of the DGGE patterns were sequenced. The bands at Day 0 revealed 97% similarity with *Acinetobacter* regardless of temperature conditions (Table 1). At Day 0, band revealed 97% similarity with *Acinetobacter bouvetii* (NR117628.1), 98% similarity with *Acinetobacter bouvetii* (NR 042234.1), and 97% similarity with *Acinetobacter beijerinckii* and *Acinetobacter venetianus* (ATCC 31012). 

On Day 7, five bands from samples from the DGGE gel were extracted and sequenced. These bands included samples from mesophilic temperature reactors (28 °C and 36 °C) and thermophilic reactors (44 °C and 52 °C). Two bands from the samples at 28 °C reactors showed 97% similarity with *Acinetobacter beijerinckii* (NR042234.1), *Acinetobacter bouvetii* (NR117628.1), and *Acinetobacter venetianus* isolates (ATCC 31012). At a higher temperature (44 °C), bands showed 100% similarity with the *Coprothermobacter proteolyticus* isolate (NR074653.1) and 95% similarity with the *Coprothermobacter platensis* isolate (NR026366.1). However, bands from 52 °C showed 100% similarity with *Tepidimicrobium ferriphilum* (NR117380.1) and *Tepidimicrobium xylanilyticum* (NR116042.1). Another band from the same reactor showed 93% similarity with *Symbiobacterium turbinis* (NR134210.1), *Symbiobacterium terraclitae* (NR134209), and *Caldibacillus debilis* (NR029016.1).

Bands from a prolonged incubation period such as Day 14 showed a slightly different set of strains. For example, on Day 14, the bands from 28 °C showed 97% similarity with *Clostridium sporosphaeroides* (NR044835.2), *Clostridium jeddahense* (NR144697.1), and *Phocea massiliensis* isolates (NR144748.1). Another band from this temperature showed 94% similarity with the *Intestinimonas butyriciproducens* isolate (NR118554.1), *Ercella succinigenes* isolate (NR134026.1), and *Papillibacter cinnamivorans* isolates (NR025025.1). At a higher temperature (44 °C), bands showed 94% similarity with the *Thermoclostridium caenicola* isolate (NR126170.1), *Intestinimonas butyriciproducens* isolate (NR118554.1), and *Hungateiclostridium thermocellum* isolate (NR074629.1). However, bands of samples at 52 °C showed 100% similarity with the *Coprothermobacter proteolyticus* isolate (NR074653.1) and 95% similarity with the *Coprothermobacter platensis* isolate (NR026366.1). Another band showed 100% similarity with the *Hungateiclostridium thermocellum* isolate (NR074629.1) and 98% similarity with *Hungateiclostridium straminisolvens* (NR024829.1). 

By the end of the experiment, there were substantial changes in the bacterial communities from all reactors. For example, on Day 60, bands from sample at 28 °C showed 87% similarity with *Galbibacter mesophilus* (NR114009.1) and *Zeaxanthinibacter enoshimensis* (NR114017.1). Another band from this temperature showed 94% similarity with *Sphaerochaeta pleomorpha* (NR102964.1), *Sphaerochaeta associata* (NR145842.1), and *Sphaerochaeta globosa* (NR114608.1). A band from this temperature also showed 93–94% similarity with *Syntrophomonas sapovorans* (NR028684.1), *Syntrophomonas curvata* (NR025752.1), and *Thermosyntropha tengcongensis* (NR109048.1). At a slightly higher temperature (36 °C), a band showed 90–91% similarity with *Sphaerochaeta pleomorpha* (NR102964.1), *Sphaerochaeta associata* strain (NR145842.1), and *Sphaerochaeta globosa* (NR114608.1). At a thermophilic temperature of 44 °C, a band showed 86% similarity with *Dielma fastidiosa* (NR125593.1), *Acholeplasma parvum* (NR042961.1), and *Acholeplasma vituli* (NR028689.1). Multiple bands were extracted from reactors operated at 52 °C. One band from this temperature showed 99% similarity with *Coprothermobacter proteolyticus* (NR074653.1), *Coprothermobacter proteolyticus* (NR029236.1), and *Coprothermobacter platensis* (NR026366.1). The other set of bands showed 94–97% similarity with *Caldicoprobacter faecalis* (NR104811.1), *Caldicoprobacter oshimai* (NR112805.1), and *Caldicoprobacter guelmensis* (NR109614.1) (Table 1). While comparing to initial stages of reactors, Day 60 results revealed that the similarity was changed significantly particularity at mesophilic conditions. Many of the isolates were not present in these reactors during the initial stages of reactions. For example, at 28 °C, *Galbibacter mesophilus* (NR114009.1) and *Zeaxanthinibacter enoshimensis* (NR114017.1) were not present during the initial stage, and at the late stage, their bands appeared.

**Table 1:** Sequence size, accession number, closest relative, and alignment similarity.

	Incubation Time				
	Day 0	Day 7	Day 14	Day 30	Day 60
Temperature	Number of extracted bands #				
28 °C	1	2	2	0	3
36 °C	1	1	0	1	1
44 °C	1	1	1	0	1
52 °C	1	2	2	4	4
Temperature	Sequence size (bp)				
28 °C	195	195	169, 172	-	188, 194, 195
36 °C	195	195	-	194	194
44 °C	195	170	171	-	194
52 °C	195	169, 194	170	172	170, 172
Temperature	Accession number				
28 °C	MK872367	MK872368	MK872373, MK872374	-	MK872383, MK872384, MK872385
36 °C	MK872367	MK872369	-	MK872378	MK872386
44 °C	MK872367	MK872370	MK872375	-	MK872387
52 °C	MK872367	MK872371, MK872372	MK872376, MK872377	MK872379, MK872380, MK872381, MK872382	MK872388, MK872389, MK872390, MK872391
Temperature		Closest relatives (alignment similarity %)			
28 °C	*Acinetobacter beijerinckii* (97%), *Acinetobacter bouvetii* (97%), *Acinetobacter venetianus* (97%)	*Acinetobacter beijerinckii* (97–98%), *Acinetobacter bouvetii* (97–98%), *Acinetobacter venetianus* (97–98%)	*Clostridium sporosphaeroides*, *Clostridium jeddahense*, *Phocea massiliensis*, *Intestinimonas butyriciproducens*, *Ercella succinigenes*, *Papillibacter cinnamivorans*		*Galbibacter mesophilus* (87%), *Zeaxanthinibacter enoshimensis* (87%), *Sphaerochaeta pleomorpha* (91%), *Sphaerochaeta associate* (90%), *Sphaerochaeta globose* (90%), *Syntrophomonas sapovorans* (94%), *Syntrophomonas curvata* (93%), *Thermosyntropha tengcongensis* (93%)
36 °C	*Acinetobacter* (97%)			*Syntrophomonas zehnderi* (92%), *Syntrophomonas sapovorans* (91%), *Syntrophomonas palmitatica* (91%)	*Sphaerochaeta pleomorpha* (91%), *Sphaerochaeta associata* (90%), *Sphaerochaeta globosa* (90%),
44 °C	*Acinetobacter* (97%)	*Coprothermobacter proteolyticus* (100%), *Coprothermobacter platensis* (95%)	*Thermoclostridium caenicola* (94%), *Intestinimonas butyriciproducens* (94%), *Hungateiclostridium thermocellum* (94%)		*Dielma fastidiosa* (86%), *Acholeplasma parvum* (86%), *Acholeplasma vituli* (86%)
52 °C	*Acinetobacter* (97%)	*Tepidimicrobium ferriphilum* (100%), *Tepidimicrobium xylanilyticum* (100%), *Symbiobacterium turbinis* (93%), *Symbiobacterium terraclitae* (92%), *Caldibacillus debilis* (89%)	*Coprothermobacter proteolyticus* (100%), *Coprothermobacter platensis* (99%), *Hungateiclostridium thermocellum* (100%)	*Caldicoprobacter faecalis* (97%), *Caldicoprobacter oshimai* (97%), *Caldicoprobacter guelmensis* (96%), *Caldicoprobacter oshimai* (96%), *Caldicoprobacter guelmensis* (96%), *Hungateiclostridium* *straminisolvens* (98%)	*Coprothermobacter proteolyticus* (99%), *Coprothermobacter platensis* (95%), *Caldicoprobacter faecalis* (97%), *Caldicoprobacter oshimai* (97%), *Caldicoprobacter guelmensis* (97%), *Caldicoprobacter faecalis* (97%)

Biological processes such as the anaerobic digestion process is one of the major processes used in wastewater and organic waste treatments, and the main purpose of utilizing these processes is to degrade the organic particles present in the waste material. Additional benefits of these processes are that they allow converting waste into value-added by-products, and these process are controlled by microbial communities present in the organic waste, and understanding their presence and activities during the anaerobic processes is crucial [24,26,27,28,29,30,31,32,33,34,35]. In anaerobic reactors, the microbial community determines the fate of anaerobic reactors in terms of biogas productions, and strong correlations between microbial communities and operational parameters are reported [36]. The DGGE-based community information provides valuable insights to assess the performance of the reactors, and such techniques can also be useful tools for evaluating the co-digestion process (i.e., digestion of feedstock, which is a mixture of manure and other organic materials such as food waste), where food waste is anaerobically co-digested with manure to produce methane [37]. Microbiomes of rumen and manure affect the performance of anaerobic reactors, and often, the abundance of *Methanobrevibacter* and *Methanoplasma* in ruminal manure enhances the biogas production and anaerobic processes [12,26,28,32,33]. In addition to these bacteria, the microbial community structures are responsible for the methanogenesis process, a crucial step for the anaerobic process, to produce methane and execute the degradation activities inside the reactors. This process is influenced by both biotic and abiotic factors, and a positive relationship between methane (CH_4_) production and phylotypes including *Methanobacteriaceae*, *Thaumarchaeota*, *Intestinibacter*, *Coprothermobacter*, and *Magnoliophyta* are reported [9,30,31,38]. 

In the current manure management strategies, the digestate of anaerobic reactors is used as a soil amendment, and soil microbial communities are influenced by the application of digestate, which has the potential to improve the productivity of soils [10]. Microbial community and their activity in the environment affect biomass degradation [38,39,40,41]. This affects the degradation rate, biogas (renewable energy) production, and retention time, which affects the size of the anaerobic reactors [6,8,42,43]. 

In order to develop an enhanced anaerobic process for faster degradation, a balanced level of microbial community is needed inside the reactors, and the DGGE-based method can assist to understand the microbial community at each step. DGGE allows the separation of the DNA fragments of the same size and with different sequences, which assists in understanding the bacterial diversity in environmental samples [4]. As an example, in order to understand the dynamics of a microbial community in swine manure during long-term storage, a previous study used the PCR-DGGE of 16S rDNA approach, and various phylotypes were identified by excising and cloning DGGE bands and comparing the 16S rDNA sequence with the sequence available in GenBank [22]. The DGGE gel technique used in this study illustrated the presence of *Clostridium butyricum*, *Clostridium disporicum*, *Pedobacter* sp., and *Rhodanobacter* sp. in swine manure. Further, the PCR-DGGE method was useful in assessing the bacterial diversity of treated and untreated milk during cold storage, and the PCR-DGGE profile showed the presence of various dominant bacteria including *Streptococcus*, *Staphylococcus*, *Pseudomonas*, *Aerococcus*, *Trichococcus floculiformis,* and *Prevotella* spp. [23]. 

It is considered that fingerprinting techniques such as DGGE provide the best compromise, when it comes to the number of samples processed, rapid and simultaneous analysis, comparison of multiple sets of samples, and the amount of information is generated [1]. Considering the number of temperature conditions and incubation periods used in this study, we utilized the potential of DGGE to understand the microbial dynamics in anaerobic digestion processes. While DGGE is a relatively time-consuming technique [2,3,4,5,22,23], it provides valuable information in terms of microbial community structures, which is essential to understand the functionality of microbial community [44]. One of the major shortcomings of the DGGE method is that it is a time-consuming and laborious process, and in order to conduct DGGE successfully, a high level of proficiency is needed to obtain separation of each band in an acrylamide gel. Further, it can only analyze small fragments up to 500 base pairs, and resolution is an issue, and DGGE is unable to separate small sequences, which cause low repeatability and reproducibility. However, integrating the PCR and DGGE-based techniques can provide much needed information to predict the performance of anaerobic reactors, which has long-term effects on the future of these systems and the economic and environmental benefits provided by these anaerobic digesters.

## 3. Conclusions

Microbial communities play a crucial role in the mesophilic and thermophilic anaerobic processes used in treating organic waste material and reducing contaminant loads. The microbial activities of these communities produce GHG gases such as methane under suitable environmental conditions. The biodiversity of the feedstock protects ecosystems of the reactors, which allows for microbial acclimatization to the continuously changing conditions inside the reactors. In this study, the focus was to enhance our existing understanding of the shifts in microbial communities during anaerobic digestion processes. During the experiments, all reactors had the same levels of organic loads and microbial community richness in the initial stages (Day 0). Temperature was varied to create various levels of stressed conditions. Under thermophilic conditions, the temperature was higher (44 °C, and 52 °C), causing a higher level of temperature stress, and under mesophilic conditions, the temperature was low (28 °C, and 36 °C), causing low-stress conditions. The experiments were prolonged to 60 Days, and microbial communities were analyzed using the PCR-DGGE approach to evaluate the dominant microbial communities. The results showed that the temperature and incubation time caused differences in microbial communities of the reactors. These microbial community structures affect the production of GHGs such as methane under anaerobic conditions from organic wastes. At the late stages of the experiments, microbial communities of the reactors were substantially changed. For example, at the thermophilic temperature of 52 °C, *Coprothermobacter proteolyticus*, a nonmotite, thermophilic non-spore forming, Gram-negative anaerobic bacterium was dominant. However, at the mesophilic temperature of 36 °C, the genus of *Sphaerochaeta* of the family *Spirochaetaceae* was abundant. The free-living anaerobic mesophilic bacteria such as *Sphaerochaeta globosa* and *Sphaerochaeta pleomorpha* were dominant at 36 °C. In the lower range of mesophilic temperatures (28 °C), the mixed communities of *Galbibacter mesophilus* (Gram-negative and rod-shaped bacterium from the genus of *Galbibacter*) and *Sphaerochaeta pleomorpha* were found. At a low thermophilic condition (44 °C), the community of *Dielma fastidiosa* (Gram-negative anaerobic rod) and *Acholeplasma parvum* were observed. This study found that molecular tools such as DGGE can be useful to investigate the diversity and the dynamics of microbial communities in a specific environment such as anaerobic reactors, and the knowledge of community composition can reveal unprecedented opportunity to understand biological GHG emissions from the environment. 

## 4. Material and Methods

### 4.1. Anaerobic Experiment and Sample Collection

**Figure 6:**
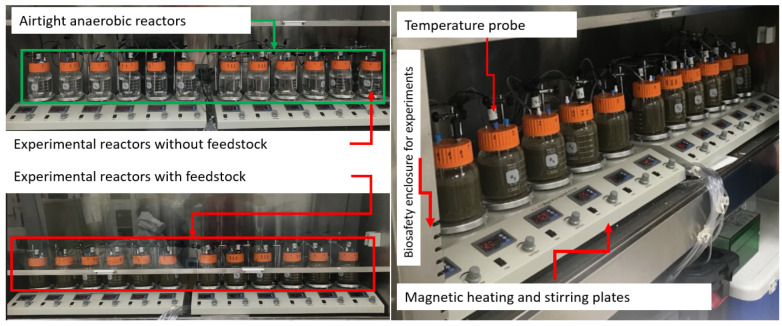
The view of anaerobic experiments designed for simulating thermophilic and mesophilic anaerobic digestion processes.

Fresh manure was collected from a full-scale dairy farm in Merced, CA, USA (herd size: 200–2200). Manure was diluted and filtered by an 850 μm standard sieve to remove large-sized particles. To design the batch anaerobic reactor, wide-mouth glass bottles were sealed with caps, and the cap was drilled to install two outlets (for sample collection and gas release). One outlet of each reactor helped to collect digested manure samples over time. Prior to starting the experiments, all reactors were tested for air leaks, and high-strength sealants and adhesives were used to design leak-free anaerobic reactors. In each reactor, an outlet was used to release the biogas formed inside the reactors. In this experiment, there were a total of 12 anaerobic reactors. Three reactors were used for designing mesophilic and thermophilic anaerobic experiments. There were 4 temperature conditions (28 °C, 36 °C, 44 °C, and 52 °C), and each temperature had 3 reactors assigned (Figure 6). Prior to starting the experiment, each reactor was filled with 500 mL of liquid manure. Similar manure was filled in three reactors for each temperature condition. Intermittent samples were collected over the incubation time, and samples were stored in −20 °C until use. Genomic DNA from these samples was extracted using the DNeasy PowerSoil Kit (Qiagen, Hilden, Germany). Sample volume of ≈2 mL was used for DNA extraction. The quality and concentration of the DNA were assessed by NanoDrop 1000 spectrophotometer (Thermo Scientific, Wilmington, DE, USA), and all extracted DNA samples were stored at −20 °C prior to PCR amplification.

### 4.2. PCR Amplification of 16S rRNA V3 Region and DGGE

The variable region V3 of the 16S rRNA was amplified using the following primers: F357-GC (5′-CGCCCGCCGCGCGCGGCGGGCGGGGCGGGGGCACGGGGGGCCTACGGGAGGCAGCAG-3′) and R518 (5′-ATTACCGCGGCTGCTGG-3′). The PCR reaction mixture contained 12.5 μL of Master Mix, 2 μL 10 mM of each primer, 16 μL of ddH_2_O, and 5 μL of template and *Taq* DNA polymerase. The temperature program consisted of initial denaturation at 95 °C for 3 min, followed by 40 cycles of 30 s at 95 °C, 30 s at 56 °C, 30 s at 72 °C, and a final extension step for 10 min at 72 °C. PCR products were evaluated using the D-Code mutation detection system (Bio-Rad, Hercules, CA, USA) for the denaturing gradient gel electrophoresis (DGGE) analysis. The DGGE technique separates DNA fragments of the same size (up to 400 bp) according to their GC content by electrophoresis. The DGGE process used acrylamide gel (16 × 20 cm) containing a linearly increasing gradient of DNA denaturants (urea and formamide). In this study, an 8% polyacrylamide gel (37.5:1 actylamide/bisactylamide) was used with a denaturing gradient ranging from 40% to 60% in which the 100% denaturing gradient was 7 mol/L urea and 40% formamide. The voltage, temperature, buffer, and time of electrophoresis were set to 180 V, 60 °C, 1 × TAE, and 4 h, respectively. After electrophoresis, the gel was washed with sterile water and placed in a SYBR Safe DNA Gel Stain (Invitrogen, USA) for 30 min, and photographs were taken under UV light. Typical bands were selected and cut from the gel, and the bands were transferred to 200 μL of sterile water in a 1.5 mL tube and incubated at 4 °C overnight. Subsequently, the tubes were centrifuged at 13,000–14,000× *g* for 10 min, and 5 μL supernatant was used as the template to re-run the PCR using primers F357 and R518. Purified PCR products were cloned into a plasmid by the TOPO TA Cloning Kit (Invitrogen, Waltham, MA, USA) and sent to the College of Biological Sciences UCDNA Sequencing Facility. ABI 3730/3730xl Capillary Electrophoresis DNA Analyzer (Applied Biosystems, Waltham, MA, USA) was used for bacterial sequencing. The sequencing results were analyzed by Basic Local Alignment Search Tool (BLAST) and compared with the National Center for Biotechnology Information (NCBI) gene library. Sequences were submitted to GenBank to obtain accession numbers. Diversity and homology of the bands were analyzed, and the system phylogenetic tree was established. The accession number and sequence size are shown in Table 1. While performing the DGGE, we used gel gradients and chemicals published elsewhere in previous studies [45,46,47]. 

### 4.3. Statistical Analysis of DGGE Images

The normalization and analysis of the patterns observed in DGGE images were performed with the BioNumerics Software version 7.6 (Applied Maths, SintMartens-Latem, Belgium). During analysis, bands with higher intensity were considered for profiling purposes. The Jaccard coefficient was used to estimate the profile similarity, and the UPGMA algorithm in the BioNumerics Software was used for cluster analysis. To estimate the DGGE profile, range-weighted richness and reflecting carrying capacity of the system were estimated as the total number of bands in each DGGE patterns. The community organization that relates the task distribution of the microbial community was estimated as the percentage of the Gini coefficient as described previously [25,36,44]. 

## Data Availability

Data are contained within the article.
